# Left Frontotemporal Region Plays a Key Role in Letter Fluency Task-Evoked Activation and Functional Connectivity in Normal Subjects: A Functional Near-Infrared Spectroscopy Study

**DOI:** 10.3389/fpsyt.2022.810685

**Published:** 2022-05-20

**Authors:** Hsin Tung, Wei-Hao Lin, Peiyuan F. Hsieh, Tsuo-Hung Lan, Ming-Chang Chiang, Yung-Yang Lin, Syu-Jyun Peng

**Affiliations:** ^1^Institute of Clinical Medicine, National Yang Ming Chiao Tung University, Taipei, Taiwan; ^2^Center of Faculty Development, Taichung Veterans General Hospital, Taichung, Taiwan; ^3^Division of Epilepsy, Neurological Institute, Taichung Veterans General Hospital, Taichung, Taiwan; ^4^College of Medicine, National Chung Hsing University, Taichung, Taiwan; ^5^Department of Psychiatry, Taichung Veterans General Hospital, Taichung, Taiwan; ^6^Department of Psychiatry, Faculty of Medicine, National Yang Ming Chiao Tung University, Taipei, Taiwan; ^7^Tsaotun Psychiatric Center, Ministry of Health and Welfare, Nantou, Taiwan; ^8^Department of Psychiatry, School of Medicine, National Yang Ming Chiao Tung University, Taipei, Taiwan; ^9^Center for Neuropsychiatric Research, National Health Research Institutes, Miaoli, Taiwan; ^10^Department of Biomedical Engineering, National Yang Ming Chiao Tung University, Taipei, Taiwan; ^11^Department of Critical Care Medicine, Taipei Veterans General Hospital, Taipei, Taiwan; ^12^Institute of Brain Science, National Yang Ming Chiao Tung University, Taipei, Taiwan; ^13^Professional Master Program in Artificial Intelligence in Medicine, College of Medicine, Taipei Medical University, Taipei, Taiwan

**Keywords:** connectivity, functional near-infrared spectroscopy (fNIRS), letter fluency task (LFT), power, verbal fluency task (VFT)

## Abstract

Letter fluency task (LFT) is a tool that measures memory, executive function, and language function but lacks a definite cutoff value to define abnormalities. We used the optical signals of functional near-infrared spectroscopy (fNIRS) to study the differences in power and connectivity between the high-functioning and low-functioning participants while performing three successive LFTs, as well as the relationships between the brain network/power and LFT performance. We found that the most differentiating factor between these two groups was network topology rather than activation power. The high-functional group (7 men and 10 women) displayed higher left intra-hemispheric global efficiency, nodal strength, and shorter characteristic path length in the first section. They then demonstrated a higher power over the left Broca's area than the right corresponding area in the latter two sections. The low-LFT group (9 men and 11 women) displayed less left-lateralized connectivity and activation power. LFT performance was only related to the network topology rather than the power values, which was only presented in the low-functioning group in the second section. The direct correlation between power and connectivity primarily existed in the inter-hemispheric network, with the timing relationship also seeming to be present. In conclusion, the high-functioning group presented more prominent left-lateralized intra-hemispheric network connectivity and power activation, particularly in the Broca's area. The low-functioning group seemed to prefer using other networks, like the inter-hemispheric, rather than having a single focus on left intra-hemispheric connectivity. The network topology seemed to better reflect the LFT performance than did the power values.

## Introduction

Multi-channel functional near-infrared spectroscopy (fNIRS) is an optical neuroimaging that can be used to study regional cortical activity. The technique is based upon so-called neurovascular coupling in which neural activity and vascular responses are closely coupled. Functional NIRS measures brain activity by detecting the hemoglobin concentration changes in the vessels of the underlying cortex. Compared with functional magnetic resonance imaging (fMRI), fNIRS is portable and provides more movement tolerance (1, 2). Moreover, it possesses better temporal resolution due to a higher sampling rate, while also recording brain signals in near real time with less time delay. Previous studies had shown that hemoglobin signals from gray matter recorded by fNIRS offered good concordance with BOLD signals of fMRI (3, 4). Owing to the characteristics described previously, the results of the task can be witnessed in real time as the fNIRS device is running. In contrast, when using fMRI to conduct tasks involving movements and speech, the performance of the participants needs to be re-recorded outside the scanner to avoid artifacts. Therefore, fNIRS has been applied during investigations of several neuropsychiatric disorders (5), including attention deficit hyperactivity disorder (6), depression (7), and schizophrenia (8).

Verbal fluency task (VFT) is a commonly used neuropsychological tool, which measures language (9), executive function (10), and memory (11). According to the properties of the cue, there are two types of VFT: categorical fluency task (CFT) and letter fluency task (LFT). CFT requires a semantic cue, which involves producing words belonging to a given category. LFT uses a phonemic hint, which involves generating words beginning with a specific syllable. Both tasks rely on the same cognitive processes, such as attention and processing speed, but they employ different searching strategies (12). Both types of VFTs had been used to screen for cognitive changes in dementia (5, 13, 14) and traumatic brain injury (15). However, in contrast to the other commonly used tools for screening cognitive function, like Mini-Mental State Examination (MMSE), these two VFTs did not have a definite cutoff score value that can be used to diagnose pathological conditions. Besides, the wide variation is also observed in the normal population.

Both VFTs depend on frontotemporal functions, particularly on the dominant side (16). Previous studies have found that the temporal lobe is of greater importance for CFT, while the frontal lobe is more important for LFT (16, 17). The LFT engages regions extending to the left rolandic operculum and left middle frontal gyrus (18). Functional NIRS showed that the CFT increased power concentrated in the left frontotemporal region (17). LFT activated the left superior and middle frontal gyri, which corresponds to the Broca's area (18). A functional MRI (fMRI) literature revealed that the left superior and anterior temporal regions were primarily activated during CFT, while the left prefrontal cortex and bilateral Broca's areas were mainly activated by LFT (19). Besides, even though declined numbers of appropriate answer and decreased concentrations of the cortical oxygenated hemoglobin had been identified in some pathological conditions (5), the definite network topology of VFTs had only partially been illustrated. The hippocampus was thought as being the component of the networks of CFT, but did not participate in the LFT networks (20). Our previous fNIRS study also identified that the ventral language pathway was used by CFT and the dorsal pathway was used by LFT (21). Measurement of concentration changes of the oxygenated hemoglobulin during VFTs by fNIRS has been found as the possible screening tool for cognitive decline (5). In dementia subjects, the oxygenated hemoglobulin concentration decreased over the right parietal region, while the concentration decreased over the frontopolar prefrontal cortexes in schizophrenia cases.

However, most studies related to VFTs and cognitions focused on the power activation. The brain network topology had not been well illustrated. Variation of performances in normal population neither been studied. Therefore, we used fNIRS to explore how the LFT network organizes in normal subjects and to identify which parameters are the key components related to their performances. Furthermore, we also analyzed the relationship between cortical activation and its connectivity.

## Materials and Methods

### Participants

The healthy subjects were retrospectively enrolled from the Departments of Neurology and Psychiatry at Taichung Veterans General Hospital during the period 2017–2018. Some were outpatients who were found to be healthy after examinations, while the others were university students studying in these departments. All were native Chinese speakers and right-handed according to the Edinburgh Handedness Inventory, without any history of psychological or neurological diseases. Subjects with any psychological problems identified by a psychiatrist, or any evidence of focal neurological deficits as determined by a neurologist, were excluded. We also excluded cases with histories of cerebrovascular disease, head trauma, or brain surgery, to remove the possible confounding effect of structural lesions. In total, 37 subjects (16 men and 21 women) were recruited. This study was approved by the Ethics Committee of Taichung Veterans General Hospital (CE18306B).

At present, there is no objective, normalized threshold for LFT scores to define the better or poorer performance. Because we only studied the trend of the LFT performance within the general and normal population, they were solely divided into the relatively better and the relatively worse groups. Because the mean and the median of the summation of LFT scores from the three sections in our study were 12.1 and 12 individually, we chose 12 as the cutoff value. Therefore, participants with a total score of 12 or less were classified as the “low-LFT” or low-functioning group, and consisted of 9 men and 11 women. The other subjects (total LFT score > 12) were classified as the “high-LFT” group or high-functioning group, and included 7 men and 10 women.

### Instrument

The hemodynamic changes over the frontotemporal regions were measured using a 52-channel NIRS instrument (ETG-4000; Hitachi Medical Co., Tokyo, Japan). The 3 × 11 cell probe with its lowest line at Fp1–Fp2 was based on the EEG international 10–20 system, and extended it laterally to T3 on the left and T4 on the right. The detailed description is mentioned in the Supplementary Material.

**Figure 1:**
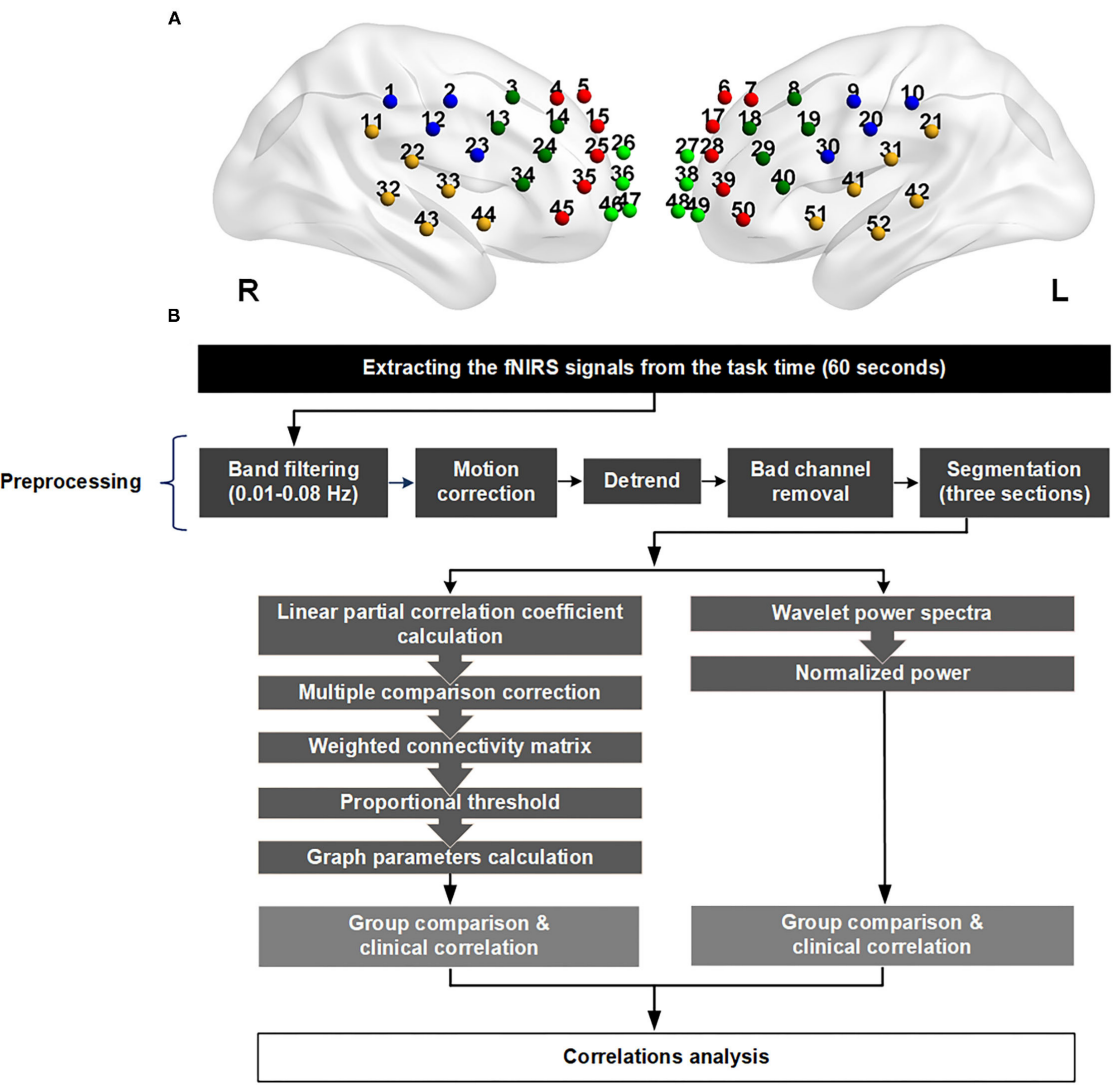
**(A)** The location of the 52 channels. The channels were divided into 11 parts according to the signals they received from the cortex. The abbreviations and channel numbers are listed below. One color represents one region in any side. Right premotor, motor, and somatosensory cortex (PMS; CH 1, 2, 12, 23): blue; left premotor, motor, and somatosensory cortex (PMS; CH 9, 10, 20, 30): blue; right Broca (CH 3, 13, 14, 24,34): dark green; left Broca (CH 8, 18, 19, 29, 40): dark green; right temporal (CH 11, 22, 32, 33, 43, 44): yellow; left temporal (CH 21, 31, 41, 42, 51, 52): yellow; right frontopolar (CH 26, 36, 46, 47): green; left frontopolar (CH 27, 38, 48, 49): green; right dorsolateral prefrontal cortex (DLPFC; CH 4,5,15,25, 35, 45): red; left dorsolateral prefrontal cortex (DLPFC; CH 6,7,17, 28, 39,50): red; midline (CH 16, 37): gray. **(B)** Flowchart of the data preprocessing and analysis. The source–detector distance of the fNIRS machine is 3.0 cm.

The 52 channels were grouped based on the standard space of the Brodmann area, and then divided into 11 cortical parcellations, depending on the signal location detected on the scalp (Figure 1A): one midline zone, as well as premotor, motor, and somatosensory cortex (PMS), Broca's area, temporal region, frontopolar cortex, and dorsolateral prefrontal cortex (DLPFC) on each side.

### Letter/Phonemic Fluency Task

All participants received the Chinese version of the letter fluency task (LFT) with a NIRS probe applied over the anterior head region. A cue using a particular Chinese syllable was given with an audible instruction, and then the participant was asked to generate as many words beginning with that syllable as possible over a span of 20 s (22). In total, the 1-min task period consisted of a series of three sections (Supplementary Figure 1), and the detailed process is described in the Supplementary Material. The number of produced words in each of the three sections of the task was recorded and summed, representing the subjects' LFT scores.

### Data Analysis

We extracted the hemoglobin signal changes during the 60-s task period and analyzed them using MATLAB R2020a (MathWorks, Natick, MA, USA). The NIRS instrument generated two types of values regarding concentration change, oxygenated hemoglobin (HbO), and deoxygenated hemoglobin (HbR). HbO reflects oxygen inflow related to brain activity, while HbR represents oxygen consumption by the tissue (23, 24). HbO has a better BOLD signal correlation and a higher signal-to-noise ratio, and is a better representative of functional connectivity than HbR (24). Therefore, we selected only HbO signals for analysis.

### Preprocessing

We discarded any channels presenting as outliers, which were deemed to be bad channels. The remaining signals were preprocessed using a 0.01–0.08 Hz band-pass filter and motion correction, and then detrended for further analysis (Supplementary Figure 2). The task period was divided into three 20-s sections, each of which began with a single phonemic cue. The values of regional activation and functional connectivity were calculated individually, and then their correlations were analyzed. The analytic flow chart is depicted in Figure 1B.

### Regional Power

To define the signal power, we constructed time-frequency spectrograms (with a 0.01-s time resolution and 10^−4^ Hz frequency resolution) of each channel through the use of complex Morlet wavelet transformation (25). The duration of the 5–10 s prior to beginning the task was defined as the baseline period. The power of the individual frequency points within the baseline period was averaged and served as the baseline power. The power during the task was then normalized by subtracting the baseline power individually. The normalized power within the time period of each 20-s section was averaged, and then averaged again by crossing the involved channels within the specific cortical parcellation. Finally, their relationship with the LFT performance and graph theory parameters of connectivity were calculated.

### Functional Connectivity

The two midline channels were excluded from the connectivity calculation because they could not be lateralized for analysis. We used the linear partial correlation coefficient to generate the r values between each of the channel pairs. The connections were selected for multiple comparison using false discovery rate (FDR) when their *p*-values were <0.05. Afterwards, the connection matrix was weighted based on their r values.

We applied proportional (sparsity-based) threshold methods for network measurement, before the network topology was quantified using graph theory. Four parameters were calculated: average node degree, average characteristic path length, global efficiency, and average nodal strength. The connections were divided into intra-hemispheric (LL and RR) and inter-hemispheric. Because there is currently no consensus on network thresholding (26), we performed the test using different cut-off values. The results of the four graph parameters are depicted in **Figures 3**–**6**. Because the proportional threshold of 0.3 had the smallest *p*-value among all parameters, we chose 0.3 as the network threshold value for subsequent correlation analysis. The detail about selecting the value for network thresholding is described in the Supplementary Material.

### Statistical Analysis

We used SPSS version 20.0 (IBM, Armonk, NY, USA) and MATLAB R2020a (MathWorks, Natick, MA, USA) for statistical analyses to study the regional power activation and network topology differences between these two groups, as well as their correlation to the LFT scores. The power of each brain region for the individual groups in every section was compared using FDR correction. All graph theory properties were compared using the two-sided Wilcoxon rank-sum test. Statistical significance was set at *p* < 0.05 for correction using multiple comparisons by FDR.

## Results

### Demographic and Clinical Data

Both groups displayed similar proportions of gender, age, and number of educational years (Table 1). The LFT scores in each section showed significant differences. The total LFT score was not correlated with age (*p* = 0.850) or educational years (*p* = 0.700) using Spearman correlation analysis (data not shown in the tables).

**Table 1:** Demographic characteristics of the Low-LFT and High-LFT groups.

	**Low-LFT (*n* = 20)**	**High-LFT (*n* = 17)**	*p*-value^*^
Age	25.0 (22.3–28.8)	26.0 (25.0–33.5)	0.311
Gender (M:F)	9:11	7:10	1.000
**Average LFT scores**
Total	9.0 (7.3–11.0)	15.0 (14.0–17.0)	<0.001
Section 1	3.0 (2.3–4.8)	6.0 (5.0–7.0)	<0.001
Section 2	3.0 (1.3–3.8)	5.0 (4–5.5)	<0.001
Section 3	3.0 (2.0–4.0)	5.0 (4–5.5)	<0.001
Education years	19.0 (16.3–20.0)	19.0 (18.0–20.0)	0.283

*Chi-square test or Mann-Whitney U test; Median (IQR).*

*LFT, letter fluency task; M, male; F, female.*

* *p < 0.05*.

### Regional Power

**Figure 2:**
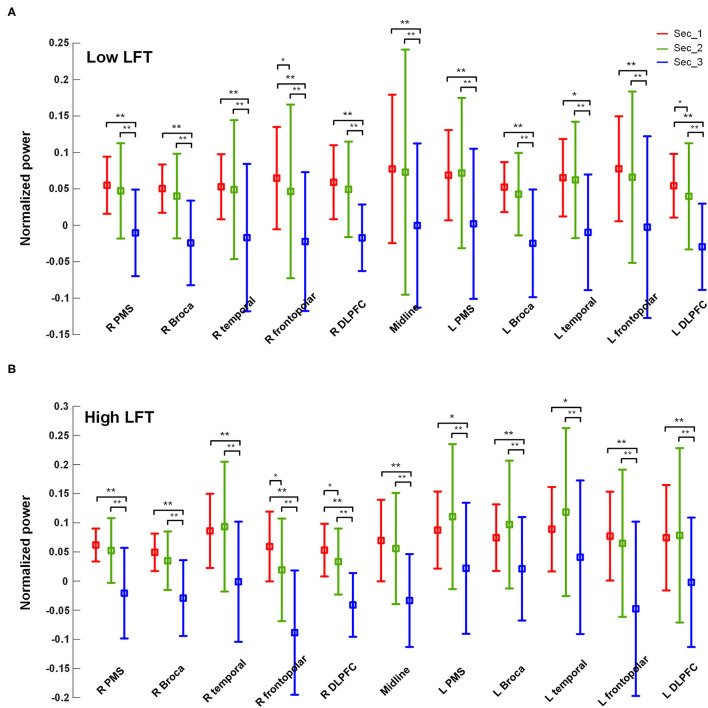
The power values of each of the brain regions within each section in the **(A)** high-LFT and **(B)** low-LFT groups.

The power activation of the 11 brain regions was calculated for each section with the results listed in Table 2. The serial power changes in the successive sections are presented in Figure 2. The power dropped dramatically in section 3 for both groups, while the power in the first two sections was relatively high. In the high-LFT group, most brain regions showed relatively higher power in the high-LFT group than that in the low-LFT group, but did not reach statistical significance. The reverse result was presented in the midline and right frontopolar regions in the second section, where the power was relatively higher in the low-LFT group than that in the high-LFT group. In the high-LFT group, the left Broca's area and left frontotemporal region showed significantly higher power than that in the right corresponding regions of the last two sections. In the low-LFT group, the power of each brain region did not show an obviously left–right asymmetrical pattern.

**Table 2:** Comparison of the power of each section between the High-LFT and Low-LFT groups.

**Region**	**Section 1**					**Section 2**					**Section 3**				
	**High LFT**	***p* ^†^**	**Low LFT**	***p* ^†^**	***p* ^‡^**	**High LFT**	***p* ^†^**	**Low LFT**	***p* ^†^**	***p* ^‡^**	**High LFT**	***p* ^†^**	**Low LFT**	***p* ^†^**	***p* ^‡^**
R_PMS	0.062 ± 0.028	0.1695	0.055 ± 0.039	0.3820	0.8820	0.052 ± 0.056	0.1488	0.047 ± 0.065	0.6165	0.8628	−0.021 ± 0.078	0.4070	−0.010 ± 0.059	0.9700	0.8628
L_PMS	0.088 ± 0.066		0.069 ± 0.062		0.8820	0.111 ± 0.124		0.027 ± 0.103		0.8628	0.022 ± 0.112		0.002 ± 0.103		0.8628
R_Broca	0.049 ± 0.032	0.1695	0.050 ± 0.033	0.5020	0.8820	0.035 ± 0.050	0.0210^*^	0.040 ± 0.058	0.9400	0.9555	−0.029 ± 0.065	0.0330^*^	−0.024 ± 0.058	0.9700	0.9555
L_Broca	0.075 ± 0.057		0.053 ± 0.034		0.8820	0.097 ± 0.110		0.043 ± 0.057		0.8628	0.021 ± 0.089		−0.025 ± 0.074		0.8628
R_temporal	0.086 ± 0.064	0.5860	0.053 ± 0.045	0.4695	0.8820	0.093 ± 0.111	0.1488	0.049 ± 0.095	0.6165	0.8628	−0.001 ± 0.103	0.1260	−0.017 ± 0.101	0.7400	0.8628
L_temporal	0.089 ± 0.072		0.065 ± 0.053		0.8820	0.118 ± 0.144		0.062 ± 0.080		0.8628	0.041 ± 0.132		−0.010 ± 0.079		0.8628
R_frontopolar	0.059 ± 0.060	0.1695	0.065 ± 0.070	0.2640	0.8820	0.019 ± 0.088	0.0620	0.047 ± 0.119	0.6165	0.9880	−0.089 ± 0.107	0.1260	−0.022 ± 0.095	0.7400	0.9880
L_frontopolar	0.077 ± 0.076		0.078 ± 0.072		0.9880	0.065 ± 0.126		0.066 ± 0.118		0.9555	−0.048 ± 0.150		−0.003 ± 0.125		0.9555
R_DLPFC	0.053 ± 0.045	0.5860	0.059 ± 0.051	0.5020	0.8820	0.034 ± 0.057	0.2660	0.049 ± 0.066	0.6165	0.9555	−0.041 ± 0.055	0.2952	−0.017 ± 0.046	0.7400	0.9555
L_DLPFC	0.074 ±0.090		0.054 ± 0.044		0.8820	0.079 ± 0.150		0.040 ± 0.073		0.8628	−0.002 ± 0.111		−0.029 ± 0.059		0.8628
midline	0.070 ± 0.070	NA	0.077 ± 0.102	NA	0.8820	0.056 ± 0.095	NA	0.073 ± 0.168	NA	0.9880	0.033 ± 0.080	NA	−0.0003 ± 0.113	NA	0.9880
Right half^a^	0.064 ± 0.041	0.1695	0.057 ± 0.040	0.3000	0.8820	0.051 ± 0.059	0.0120^*^	0.048 ± 0.067	0.7212	0.9555	−0.033 ± 0.059	0.0240^*^	−0.018 ± 0.051	0.9700	0.9555
Left half^b^	0.080 ± 0.060		0.063 ± 0.044		0.8820	0.094 ± 0.103		0.055 ± 0.064		0.8628	0.009 ± 0.090		−0.014 ± 0.056		0.8628
All power^c^	0.072 ± 0.047	NA	0.060 ± 0.041	NA	0.8820	0.072 ± 0.078	NA	0.052 ± 0.063	NA	0.8628	−0.013 ± 0.072	NA	−0.015 ± 0.048	NA	0.8628

† *Wilcoxon signed rank test, comparing the powers of the corresponding regions in the left and the right hemispheres, after multiple comparison corrections within each section.*

‡ *Wilcoxon signed rank test, comparing the powers of the High-LFT and Low-LFT groups in each section, after multiple comparison corrections within each section.*

* *p <0.05, when the difference present is between the left and the right corresponding regions after multiple comparisons using FDR.*

*NA, not available.*

a *Average power of the 25 channels located over the right fronto-temporal region.*

b *Average power of the 25 channels located over the left fronto-temporal region.*

c *Average power of the all the 52 channels (including midline channels).*

*PMS, premotor, motor, and somatosensory cortex; DLPFC, dorsolateral prefrontal cortex (DLPFC)*.

### Functional Connectivity

**Figure 3:**
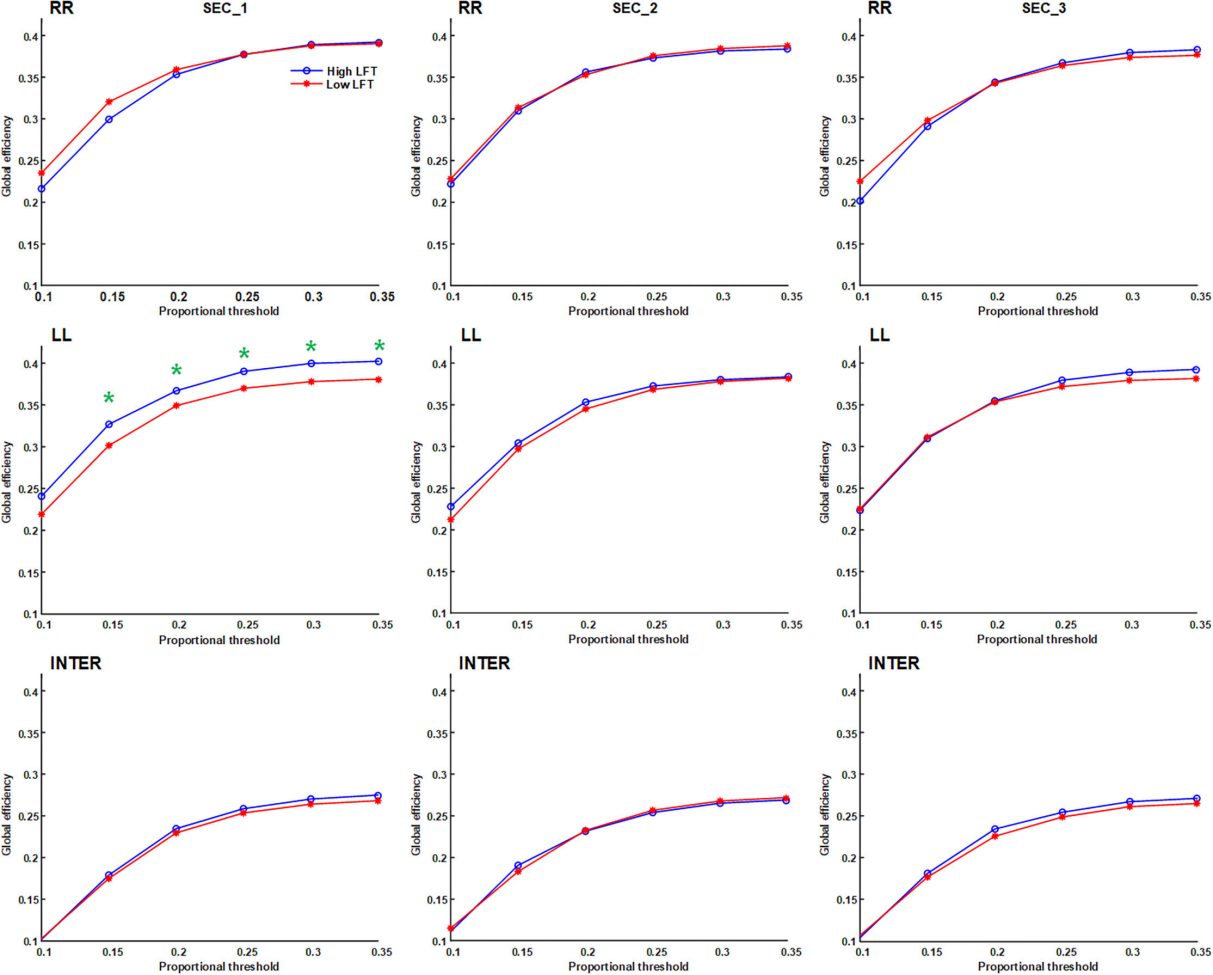
Global efficiency of the inter-hemispheric and the inter-hemispheric networks in each section using different proportional threshold values (*p < 0.05).

**Figure 4:**
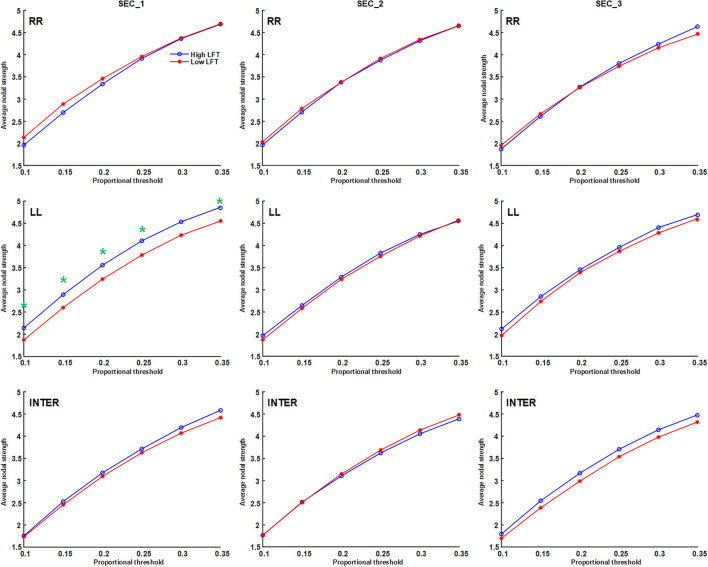
Average nodal strength of the inter-hemispheric and the inter-hemispheric networks in each section using different proportional threshold values (*p < 0.05).

**Figure 5:**
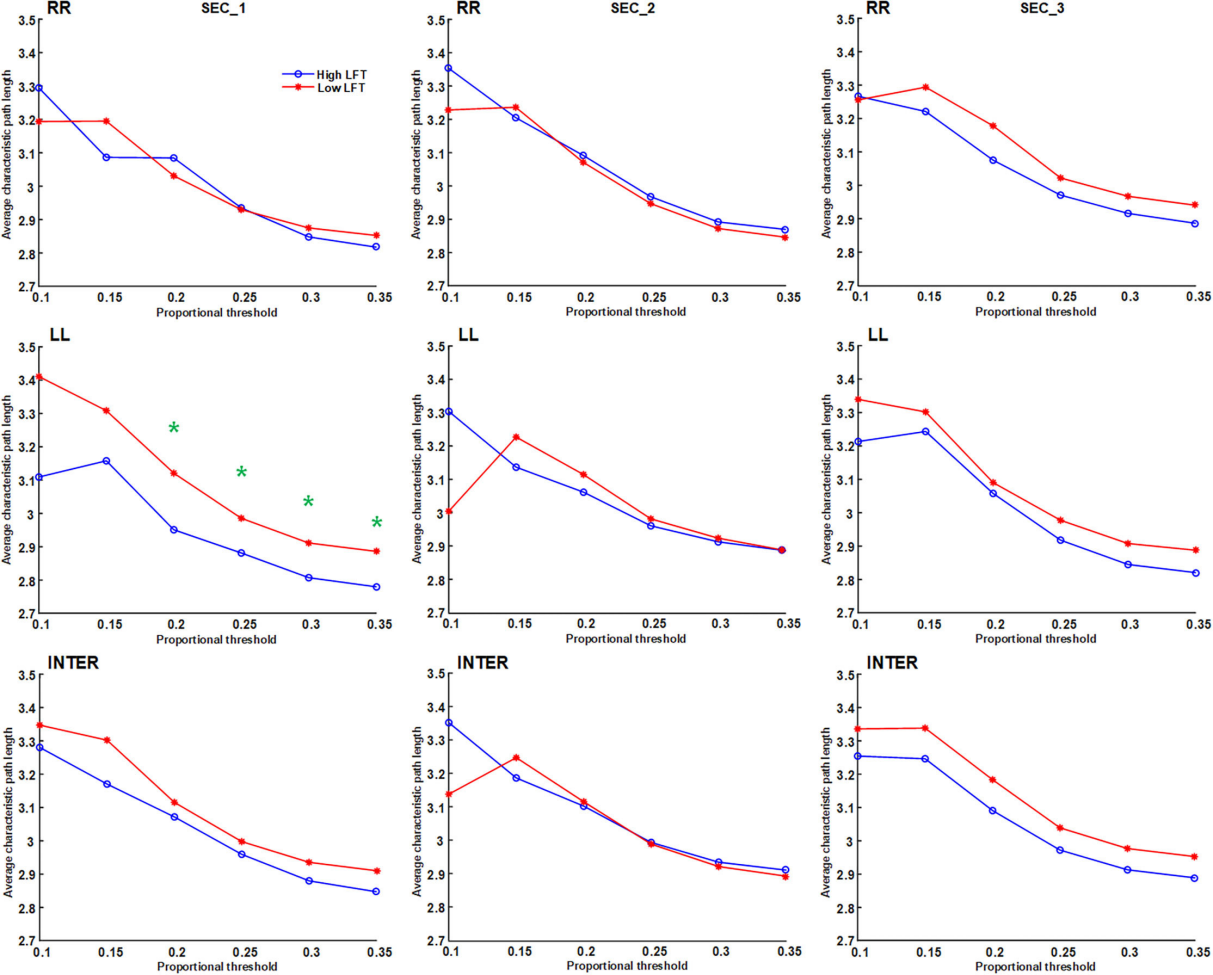
Average characteristic path length of the inter-hemispheric and the inter-hemispheric networks in each section using different proportional threshold values (*p < 0.05).

**Figure 6:**
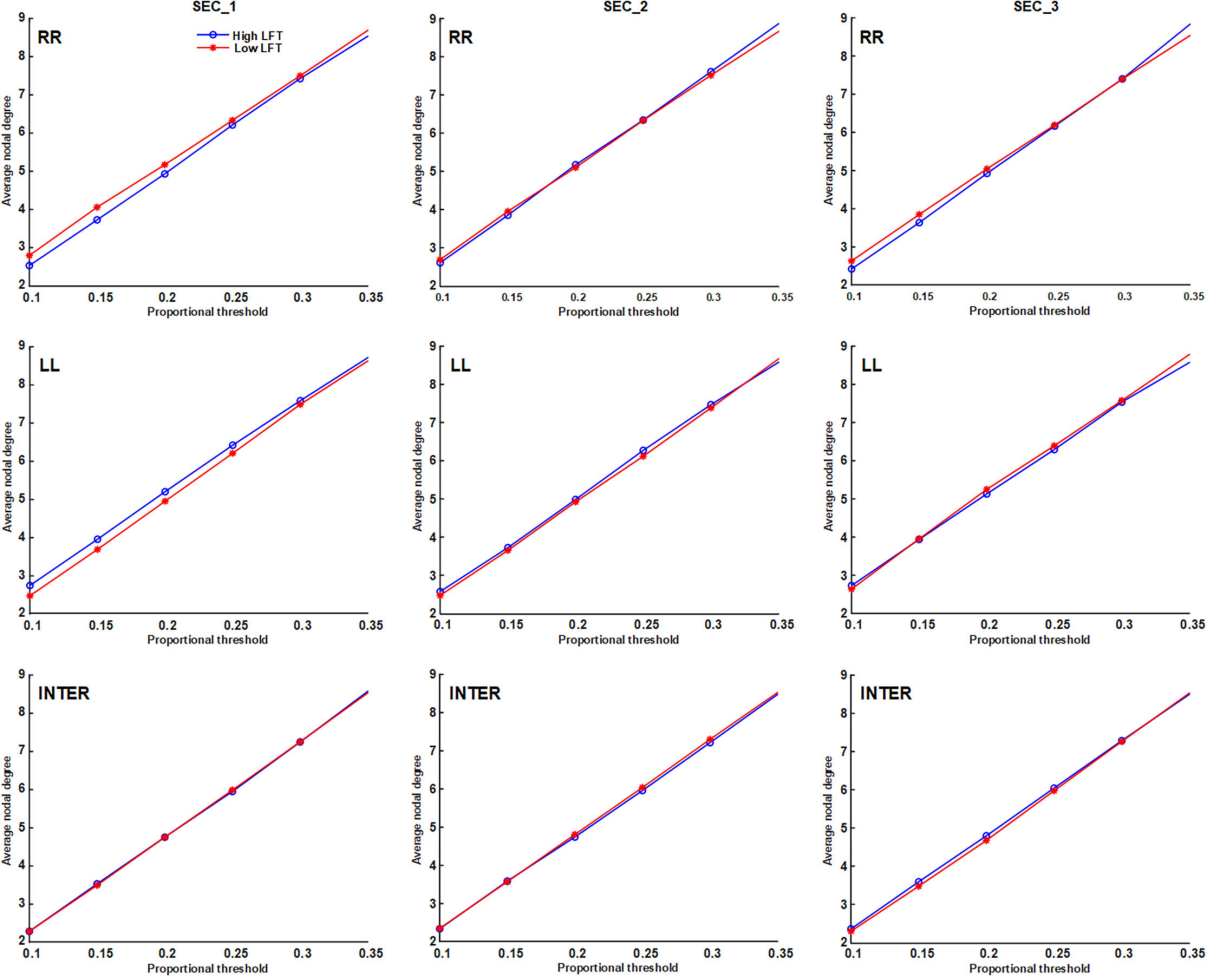
Average node degree of the inter-hemispheric and the inter-hemispheric networks in each section using different proportional threshold values.

The network topology was constructed using the proportional threshold with the value of 0.3. The high-LFT group showed higher left intra-hemispheric global efficiency (Figure 3), higher average nodal strength (Figure 4), and shorter average characteristic path length (Figure 5) only in the first section. However, the average nodal degree did not show the differences (Figure 6). The RR intra-hemispheric and inter-hemispheric networks showed almost identical topology in these two groups.

### LFT Performance Correlation

The sum of the LFT scores were calculated before their relationship with the corresponding network topology was surveyed. The regional brain power for the three sections did not show any obvious correlation with the sum of the LFT scores (Supplementary Table 1). However, in terms of connectivity, the total LFT scores were only related to the parameters in the second section of the low-LFT group. They were negatively correlated with the LL intra-hemispheric and inter-hemispheric average characteristic path length, and positively correlated with both sides of the intra-hemispheric global efficiency, as well as the left intra-hemispheric average nodal strength (Table 3).

**Table 3:** Correlation between the sum LFT scores and graph theory parameters using the proportional threshold 0.3.

		**Section 1**		**Section 2**		**Section 3**	
		**Low LFT^†^**	**High LFT^†^**	**Low LFT^†^**	**High LFT^†^**	**Low LFT^†^**	**High LFT^†^**
Average node degree	RR	0.0905 (0.7126)	0.1243 (0.6466)	−0.0233 (0.9246)	0.0208 (0.9391)	0.0905 (0.7126)	−0.2026 (0.4518)
	LL	−0.3161 (0.1874)	−0.0374 (0.8905)	0.1707 (0.4848)	−0.0517 (0.8492)	−0.3161 (0.1874)	0.1921 (0.4761)
	INTER	0.2052 (0.3994)	−0.1883 (0.4850)	−0.2027 (0.4054)	−0.1577 (0.5597)	0.2052 (0.3994)	−0.1424 (0.5989)
Average characteristic path length	RR	0.1262 (0.6066)	0.1810 (0.5022)	−0.4194 (0.0739)	−0.0465 (0.8643)	−0.2221 (0.3608)	0.4170 (0.1081)
	LL	0.0165 (0.9464)	0.1661 (0.5387)	−0.4743 (0.0402)^*^	0.1830 (0.4976)	0.1446 (0.5548)	0.0885 (0.7446)
	INTER	−0.0107 (0.9653)	0.1323 (0.6253)	−0.4632 (0.0458)^*^	0.0400 (0.8832)	−0.0541 (0.8260)	0.2637 (0.3237)
Global efficiency	RR	−0.1932 (0.4281)	−0.0103 (0.9698)	0.4577 (0.0488)^*^	0.1005 (0.7112)	0.1997 (0.4125)	−0.4008 (0.1240)
	LL	−0.0991 (0.6864)	−0.0543 (0.8418)	0.4988 (0.0297)^*^	−0.1139 (0.6746)	−0.1474 (0.5472)	−0.1989 (0.4602)
	INTER	−0.0881 (0.7199)	−0.0566 (0.8352)	0.4295 (0.0665)	−0.2622 (0.3266)	0.1751 (0.4733)	−0.2846 (0.2854)
Average nodal strength	RR	−0.0106 (0.9655)	0.1005 (0.7112)	0.4167 (0.0760)	0.0925 (0.7334)	0.1971 (0.4187)	−0.3875 (0.1381)
	LL	−0.2111 (0.3856)	−0.0226 (0.9337)	0.5019 (0.0286)^*^	−0.0296 (0.9135)	−0.2258 (0.3527)	−0.1140 (0.6741)
	INTER	0.1243 (0.6121)	0.0087 (0.9744)	0.3578 (0.1326)	0.0771 (0.7764)	0.1318 (0.5907)	−0.2552 (0.3402)

† *Spearman partial correlations (p-value), with age as the covariant.*

*RR, LL, intra-hemispheric connections in right or left side; INTER, inter-hemispheric connections.*

* *p < 0.05*.

### Relationship Between Power and Connectivity

In the high-LFT group, only inter-hemispheric average nodal strength was negatively associated with the average power of 25 channels over the left frontotemporal regions in the first section (Table 4). In the low-LFT group, the correlation was only present in the third section (Table 5). The average power of the left half channels was positively correlated with the inter-hemispheric average node degree, global efficiency, and average nodal strength. However, the average power of the right half channels was negatively correlated with the LL intra-hemispheric average node degree.

**Table 4:** Correlation between power and connectivity in the High-LFT group.

		**Left power**			**Right power**		
		**Sec 1**	**Sec 2**	**Sec 3**	**Sec 1**	**Sec 2**	**Sec 3**
Average node degree	RR	−0.155 (0.554)	−0.043 (0.870)	−0.028 (0.914)	−0.103 (0.694)	−0.105 (0.689)	0.037 (0.888)
	LL	0.383 (0.130)	0.025 (0.925)	−0.314 (0.220)	0.097 (0.711)	−0.007 (0.978)	−0.134 (0.609)
	INTER	−0.168 (0.518)	0.213 (0.411)	0.396 (0.116)	0.093 (0.722)	0.317 (0.215)	0.218 (0.400)
Average characteristic path length	RR	0.365 (0.149)	0.157 (0.548)	−0.179 (0.492)	0.265 (0.305)	0.083 (0.751)	−0.132 (0.613)
	LL	0.044 (0.866)	0.061 (0.815)	−0.113 (0.667)	0.284 (0.269)	−0.025 (0.926)	−0.306 (0.232)
	INTER	0.458 (0.064)	0.314 (0.220)	−0.203 (0.434)	0.390 (0.122)	0.238 (0.358)	−0.284 (0.269)
Global efficiency	RR	−0.433 (0.083)	−0.356 (0.161)	0.143 (0.583)	−0.432 (0.084)	−0.266 (0.302)	0.141 (0.589)
	LL	−0.072 (0.782)	−0.155 (0.554)	0.121 (0.643)	−0.258 (0.318)	−0.070 (0.790)	0.330 (0.196)
	INTER	−0.433 (0.082)	−0.098 (0.707)	0.413 (0.100)	−0.354 (0.163)	−0.022 (0.933)	0.346 (0.173)
Average nodal strength	RR	−0.412 (0.101)	−0.245 (0.343)	0.130 (0.619)	−0.404 (0.107)	−0.179 (0.492)	0.123 (0.639)
	LL	0.110 (0.673)	−0.137 (0.599)	0.105 (0.687)	−0.108 (0.680)	−0.100 (0.701)	0.331 (0.195)
	INTER	−0.495 (0.043)^*^	−0.348 (0.171)	0.397 (0.115)	−0.326 (0.202)	−0.284 (0.269)	0.358 (0.158)

*Spearman correlation: Spearman's ρ (p-value).*

* *p <0.05.*

*RR, LL, intra-hemispheric connections in right or left side; INTER, inter-hemispheric connections*.

**Table 5:** Correlation between power and connectivity in the Low-LFT group.

		**Left power**			**Right power**		
		**Sec 1**	**Sec 2**	**Sec 3**	**Sec 1**	**Sec 2**	**Sec 3**
Average node degree	RR	0.072 (0.762)	−0.132 (0.580)	−0.101 (0.671)	0.042 (0.860)	−0.140 (0.556)	0.172 (0.468)
	LL	−0.137 (0.563)	0.186 (0.432)	−0.393 (0.087)	−0.211 (0.373)	−0.014 (0.954)	−0.468 (0.037)^*^
	INTER	0.032 (0.895)	0.165 (0.486)	0.497 (0.026)^*^	0.094 (0.693)	0.375 (0.103)	0.306 (0.189)
Average characteristic path length	RR	−0.200 (0.398)	0.259 (0.269)	−0.298 (0.202)	−0.120 (0.613)	0.272 (0.245)	−0.200 (0.398)
	LL	−0.316 (0.175)	0.029 (0.905)	0.023 (0.922)	−0.213 (0.368)	0.147 (0.535)	0.310 (0.184)
	INTER	−0.320 (0.169)	0.273 (0.244)	−0.433 (0.056)	−0.211 (0.373)	0.305 (0.192)	−0.156 (0.510)
Global efficiency	RR	0.275 (0.240)	−0.253 (0.282)	0.192 (0.418)	0.166 (0.486)	−0.199 (0.399)	0.139 (0.560)
	LL	0.198 (0.403)	−0.167 (0.482)	−0.050 (0.833)	0.093 (0.696)	−0.290 (0.214)	−0.194 (0.412)
	INTER	0.429 (0.059)	−0.202 (0.394)	0.500 (0.025)^*^	0.363 (0.116)	−0.281 (0.230)	0.207 (0.381)
Average nodal strength	RR	−0.038 (0.875)	−0.195 (0.409)	0.134 (0.574)	0.164 (0.490)	−0.212 (0.369)	0.235 (0.319)
	LL	−0.197 (0.405)	−0.078 (0.743)	−0.092 (0.701)	0.092 (0.701)	−0.198 (0.402)	−0.280 (0.232)
	INTER	−0.033 (0.890)	−0.304 (0.193)	0.559 (0.010)^*^	0.323 (0.164)	−0.305 (0.191)	0.287 (0.220)

*Spearman correlation: Spearman's ρ (p-value).*

* *p < 0.05.*

*RR, LL, intra-hemispheric connections in right or left side; INTER, inter-hemispheric connections*.

## Discussion

This is the first study using fNIRS for establishing the relationship among the frontotemporal connectivity, the regional power activation, and the LFT performance.

The frontal lobe plays an important role in “switching” (27), which is the important component in phonemic word generation, allowing for flexibility during lexical access (27). Moreover, fNIRS studies have revealed relatively greater augmentation of frontal activation when performing LFT as compared with CFT (22, 28). The channels of the fNIRS instrument used in this study mostly covered the frontal and anterior temporal regions, which allowed for a more reliable signal detection of LFT than that of CFT.

A previous fMRI study had demonstrated that both young and old healthy participants had overlapping and different LFT activation patterns (29). The clusters activated in the younger group occurred in the left Broca's area (Brodmann 45), left superior temporal gyrus (Brodmann 22), and left inferior frontal gyrus (Brodmann 9), whereas the group also had relatively higher LFT scores. This result was similar to our findings in the high-LFT group, where the left Broca's area had significantly higher power when compared with the right corresponding region. In the aged group, additional significant clusters were detected in the right frontopolar area (Brodmann 10). However, their participants were older than our low-LFT group cases (64–88 years old vs. 17–53 years old), even though both groups had relatively lower LFT scores. These two results were not closely comparable. Relatively higher power over the midline, as well as the left frontopolar and temporal regions was also noted in our low-LFT group, even though it did not reach statistical difference. Such phenomenon suggests that better LFT performance is primarily related to activation of the left Broca's area. Our high-functioning group cases showed a more prominent left lateralization activation pattern than the low-functioning group. We suggest those who were unable to concentrate firmly to activate the Broca's area showed lower LFT scores. This further indicates that activation of the frontopolar, temporal, and midline areas serves as an alternative pathway and is related to weaker competence.

The regions with relatively higher power had been reported as having roles in language production and executive function in previous literatures. The Broca's area of the dominant hemisphere manages language production and has also been reported to be the primary activated area when performing LFT (17). The temporal area is a part of the ventral language pathway according to the dual language stream hypothesis, and mediates lexical concepts and comprehension (30). The midline channels represent the frontopolar region, which integrates cognitive information to re-disperse activity toward the action (31). Bilateral frontopolar activation has been reported when LFT is carried out (32).

The power reflects the amount of regional neuronal activation, and the connectivity indicates the correlations between frequency fluctuations in the separate brain regions. In our study, the strongest power occurred in the first two sections of both groups. Power dropped dramatically in the third section, where most of the values were lower than those in the baseline values. This phenomenon was also noted in previous fMRI studies and was interpreted as the “practice effect” (33–35). Repetition of working memory tasks attenuates activation, which is thought to be due to an improvement in efficiency, where the behavior of the performance is maintained. Another explanation is that persistent activation-related exhaustion of oxygenated hemoglobin possibly occurs, as according to the results of a previous fNIRS study (36).

Network thresholding assists in the elimination of spurious connections, which in turn strengthens the characteristics of the network topology (26). We used proportional thresholds for network measurements with step-wise increasing values. Although their values regarding the graph theory parameters differed, the results showed a consistent trend. The main differences between the high-LFT and low-LFT groups manifested in the first section, and presented similar in the following two sections. The most differentiating factor between the high-LFT and low-LFT groups was network topology rather than the activation power. High-functional cases displayed higher global efficiency and nodal strength, as well as shorter path length in the first section. This suggests that the initial network topology is more asymmetrical and more left-lateralized in high-functioning LFT, even becoming similar in the second and the third sections.

Our healthy participants demonstrated a wide distribution of LFT performance, which was quantified by their total LFT scores, but not related to age or educational years. Most left intra-hemispheric parameters of the second section were related to LFT performance only in the low-functioning group. In addition, some right intra-hemispheric and inter-hemispheric network topologies in the second section were also related to their LFT performance. Therefore, we suppose that the low-functioning group dispersed their LFT network into other pathways, instead of only the left intra-hemispheric connectivity, which decreases LFT competency. This result also suggests that the cognitive and executive function in normal population may be better interpreted by the network topology rather than the power activation, like dementia and schizophrenia (5).

In addition, we even found that high-LFT groups had the higher efficient and dense network topology in the first section, as well as higher power over both the Broca's area and the left frontotemporal region in the last two sections. Neither power nor current network parameters had directly reflected the LFT scores in the high-functioning group. We speculate that network connectivity of the left hemisphere used by the high-LFT group was more concentrated in a small area, like the Broca's area. Its effect may be diluted by using the graph theory when the whole left frontotemporal area was counted.

The network topology in our study showed similar inter-hemispheric and intra-hemispheric values, and presented almost the same throughout the three sections. The only exception was in global efficiency, whose values were relatively lower in the inter-hemispheric than intra-hemispheric aspects during all three sections (Figure 3), regardless of the thresholding values used. Inter-hemispheric global efficiency was only one half of intra-hemispheric connectivity. This suggests that performance of the letter fluency task primarily depends on the intra-hemispheric network, and less on inter-hemispheric connectivity (21), which is reflected in their use of the dorsal language pathway (19).

The changes in power and connectivity were not parallel. The relationship between the left frontotemporal power and their functional connectivity mostly existed in the inter-hemispheric networks of both groups. In the high-functioning group, higher inter-hemispheric nodal strength was related to lower left-side power, which indicates that the left frontotemporal region possesses sufficient efficiency and requires less additional efforts in the first section. In the low-functioning group, the higher left-side power was associated with higher inter-hemispheric nodal degree, nodal strength, and global efficiency in the third section. This suggests that considerable efforts and supports from left frontotemporal activation are needed to maintain the average level of inter-hemispheric connections. In addition, the right frontotemporal power required less activation when the left intra-hemispheric nodal degree was achieved in the third section. Therefore, this indirectly echoes that the left frontotemporal region remains the important area for LFT. Low-functioning cases also used less left intra-hemispheric but relatively more inter-hemispheric connectivity to perform LFT.

Some fMRI studies have reported that task-evoked activation and connectivity are not usually correlated (37–39), and a fNIRS study also found a similar outcome (40). These results depended on the parcellation of brain regions and the analytic methods that were employed. The connectivity was still present even when the activation pattern was not evident (37), which was also observed in our study. The network topology was maintained in the third section, although the power had dropped. One study discovered that the more activated brain regions exhibited significantly greater connectivity changes than those seen in the non-activated regions (39). The authors concluded that hubs and activation patterns modify the network topology (39). Our study found the possible timing relationship between the connectivity and the power activation. The left intra-hemispheric nodal strength and efficiency increased initially during the first section in the high-functioning group, with the power of the left Broca's area asymmetrically increasing in the latter two sections. This disclosed the elevated power that could result from an increase in network efficiency and nodal strength. However, to firmly establish the definite relationship between power and connectivity, further studies must be explored.

Our studies had some limitations. First, only cortical neuronal signals could be detected, while signals from deep structures could not be obtained. This is an inevitable shortcoming of the fNIRS instrument. Second, only the frontal lobes and part of the temporal lobes could be covered by the electrodes. As a result, signals from the posterior head region might have been missed. Third, our data were collected retrospectively and the sample size was relatively small. Finally, no other comprehensive neuropsychiatric examinations were conducted. Therefore, further studies involving a larger sample size are still necessary.

## Conclusion

The performance of LFT varies greatly even in a normal population. The activation power surged in the first two sections and dropped dramatically during the third. The main differentiating factor between high-functioning and low-functioning groups was network topology rather than activation power. The high-functional group displayed higher left intra-hemispheric global efficiency and nodal strength, but decreased characteristic path length in the first section, which suggested a greater left lateralized connectivity pattern in the high-LFT group. In addition, the high-LFT group demonstrated higher power over the left Broca's area than it did in the right corresponding area in the latter two sections. The low-LFT group displayed less left-lateralized connectivity and activation power, which suggested other networks were used by them, including inter-hemispheric connectivity, rather than just the single left intra-hemispheric network.

The network topology seemed to better reflect the LFT performance than the power values. We found that the well-constructed left intra-hemispheric connectivity and the well-activated left Broca's area seemed to be related to the more excellent LFT performance.

## Data Availability Statement

The data analyzed in this study is subject to the following licenses/restrictions: the original datasets are inquiries can be directed to the corresponding author. Requests to access these datasets should be directed to S-JP, sjpeng2019@tmu.edu.tw.

## Ethics Statement

This study was approved by the Ethics Committee of Taichung Veterans General Hospital (CE18306B). Written informed consent for participation was not required for this study in accordance with the national legislation and the institutional requirements.

## Author Contributions

HT and S-JP: work conception, study design, and final approval of the version. W-HL, T-HL, and PH: examination enrollment. W-HL: conducting NIRS examination. S-JP: data processing and analysis. HT: clinical data analysis and drafting the work. HT, T-HL, M-CC, and Y-YL: interpretation of data. Y-YL and S-JP: revising the work for valuable intellectual content. All authors contributed to the article and approved the submitted version.

## Funding

This study was supported by a grant from the Ministry of Science and Technology, Taiwan, under the project MOST 110-2221-E-038-008 and MOST 108-2314-B-075A-013 and by a grant from the Higher Education Sprout Project by the Ministry of Education, Taiwan.

## Conflict of Interest

The authors declare that the research was conducted in the absence of any commercial or financial relationships that could be construed as a potential conflict of interest.

## Publisher's Note

All claims expressed in this article are solely those of the authors and do not necessarily represent those of their affiliated organizations, or those of the publisher, the editors and the reviewers. Any product that may be evaluated in this article, or claim that may be made by its manufacturer, is not guaranteed or endorsed by the publisher.
