# Supplementary material for: Left Frontotemporal Region Plays a Key Role in Letter Fluency Task-Evoked Activation and Functional Connectivity in Normal Subjects: A Functional Near-Infrared Spectroscopy Study
Source: Front Psychiatry. 2022 May 20;13:810685. doi: 10.3389/fpsyt.2022.810685 (PMC9205401; doi:10.3389/fpsyt.2022.810685)
Supplement: Supplementary file 1 [file Table_1.docx]

**Supplementary Table 1. The relationship between the power of each brain region and LFT performance.**

| **Region** | **Section 1** | | **Section 2** | | **Section 3** | |
| --- | --- | --- | --- | --- | --- | --- |
|  | High LFT | Low LFT | High LFT | Low LFT | High LFT | Low LFT |
| R_PMS | 0.095 (0.717) | -0.104 (0.661) | 0.477 (0.053) | -0.331 (0.154) | -0.463 (0.062) | -0.327 (0.159) |
| L_PMS | -0.319 (0.212) | 0.127 (0.595) | -0.165 (0.527) | 0.037 (0.877) | -0.177 (0.498) | -0.119 (0.618) |
| R_Broca | -0.177 (0.497) | 0.159 (0.503) | 0.133 (0.611) | -0.226 (0.338) | -0.428 (0.086) | -0.025 (0.917) |
| L_Broca | 0.055 (0.834) | 0.011 (0.964) | 0.075 (0.774) | 0.048 (0.839) | -0.346 (0.174) | -0.179 (0.451) |
| R_temporal | -0.264 (0.306) | 0.091 (0.704) | 0.171 (0.511) | -0.242 (0.303) | -0.306 (0.232) | -0.294 (0.209) |
| L_temporal | -0.364 (0.151) | 0.030 (0.900) | -0.079 (0.762) | -0.110 (0.644) | -0.198 (0.446) | -0.207 (0.381) |
| R_frontopolar | -0.097 (0.710) | 0.168 (0.478) | -0.171 (0.511) | -0.131 (0.583) | -0.174 (0.504) | -0.306 (0.189) |
| L_frontopolar | -0.211 (0.415) | 0.233 (0.344) | -0.098 (0.707) | -0.005 (0.985) | -0.230 (0.375) | -0.085 (0.721) |
| R_DLPFC | -0.051 (0.845) | 0.154 (0.518) | 0.201 (0.440) | 0.086 (0.718) | -0.288 (0.262) | -0.102 (0.668) |
| L_DLPFC | -0.041 (0.876) | -0.030 (0.900) | 0.153 (0.557) | -0.193 (0.415) | -0.419 (0.094) | -0.161 (0.498) |
| midline | -0.295 (0.251) | 0.015 (0.949) | -0.075 (0.774) | 0.205 (0.387) | -0.311 (0.224) | -0.153 (0.519) |

**Spearman correlation:** Spearman's ρ (p value); L = left; R = right; PMS = premotor, motor, and somatosensory cortex; DLPFC = dorsolateral prefrontal cortex (DLPFC).
