# Supplementary material for: Left Frontotemporal Region Plays a Key Role in Letter Fluency Task-Evoked Activation and Functional Connectivity in Normal Subjects: A Functional Near-Infrared Spectroscopy Study
Source: Front Psychiatry. 2022 May 20;13:810685. doi: 10.3389/fpsyt.2022.810685 (PMC9205401; doi:10.3389/fpsyt.2022.810685)
Supplement: Supplementary file 2 [file Table_2.docx]

**Supplementary Table 2. Channel location of our near-infrared spectroscopy.**

|  | **MNI coordinate position** | | | **Side** |
| --- | --- | --- | --- | --- |
| **Channel** | **x** | **y** | **z** |  |
| 1 | 64.93281 | -28.0671 | 42.399116 | R |
| 2 | 59.70503 | -3.23792 | 42.542505 | R |
| 3 | 47.78253 | 22.50822 | 44.137349 | R |
| 4 | 32.58769 | 40.79643 | 43.705199 | R |
| 5 | 12.59195 | 51.98963 | 44.611616 | R |
| 6 | -10.4995 | 52.17602 | 44.483318 | L |
| 7 | -30.408 | 41.21593 | 43.573699 | L |
| 8 | -45.986 | 23.27554 | 43.880078 | L |
| 9 | -57.145 | -1.33484 | 42.847407 | L |
| 10 | -63.3235 | -25.4495 | 42.205116 | L |
| 11 | 67.0516 | -35.6534 | 29.940658 | R |
| 12 | 66.46264 | -10.4511 | 31.011984 | R |
| 13 | 57.70924 | 16.43627 | 31.398379 | R |
| 14 | 44.35397 | 40.93354 | 32.060983 | R |
| 15 | 24.38628 | 57.52073 | 32.171181 | R |
| 16 | 2.147989 | 60.26062 | 31.958525 | Midline |
| 17 | -22.4491 | 57.2198 | 32.438885 | L |
| 18 | -41.999 | 41.91954 | 31.666899 | L |
| 19 | -55.2457 | 17.54767 | 31.397178 | L |
| 20 | -64.4502 | -8.21472 | 31.332079 | L |
| 21 | -66.3986 | -33.6005 | 30.376855 | L |
| 22 | 68.37563 | -19.1502 | 17.528016 | R |
| 23 | 63.78725 | 7.844551 | 20.209881 | R |
| 24 | 53.61845 | 35.83654 | 20.054129 | R |
| 25 | 37.0564 | 57.4121 | 19.917214 | R |
| 26 | 14.50847 | 68.2837 | 21.274487 | R |
| 27 | -12.7902 | 67.7976 | 20.112237 | L |
| 28 | -35.0793 | 57.60609 | 20.269782 | L |
| 29 | -51.746 | 36.2857 | 19.138362 | L |
| 30 | -61.5607 | 9.53264 | 19.965549 | L |
| 31 | -67.3221 | -16.8367 | 18.819988 | L |
| 32 | 70.75545 | -29.1383 | 2.197666 | R |
| 33 | 65.90226 | -4.08694 | 5.411163 | R |
| 34 | 58.78277 | 26.73609 | 8.084923 | R |
| 35 | 46.56024 | 52.09218 | 7.152344 | R |
| 36 | 26.83951 | 67.93374 | 8.511992 | R |
| 37 | 2.362246 | 68.60508 | 8.158449 | Midline |
| 38 | -23.9001 | 68.08626 | 8.511195 | L |
| 39 | -44.3199 | 52.8298 | 6.27344 | L |
| 40 | -56.5689 | 28.05184 | 7.133363 | L |
| 41 | -63.7712 | -1.51474 | 6.141245 | L |
| 42 | -69.1282 | -27.4722 | 1.396733 | L |
| 43 | 69.11361 | -13.0674 | -10.337243 | R |
| 44 | 59.612 | 10.60029 | -8.323611 | R |
| 45 | 52.92638 | 42.93437 | -5.831591 | R |
| 46 | 38.14588 | 63.22186 | -4.162938 | R |
| 47 | 14.83446 | 70.78854 | -2.620499 | R |
| 48 | -12.8134 | 71.54969 | -2.771357 | L |
| 49 | -35.0671 | 63.39415 | -4.40196 | L |
| 50 | -50.8984 | 44.54615 | -6.192143 | L |
| 51 | -57.3635 | 14.31495 | -7.758785 | L |
| 52 | -68.2962 | -11.467 | -11.968529 | L |

R = Right; L = Left.
