# Supplementary material for: Left Frontotemporal Region Plays a Key Role in Letter Fluency Task-Evoked Activation and Functional Connectivity in Normal Subjects: A Functional Near-Infrared Spectroscopy Study
Source: Front Psychiatry. 2022 May 20;13:810685. doi: 10.3389/fpsyt.2022.810685 (PMC9205401; doi:10.3389/fpsyt.2022.810685)
Supplement: Supplementary file 3 [file Table_3.docx]

**Supplementary Table 3. The detail characteristics of the subjects.**

| **Code** | **group** | **Gender** | **Age** | **Education year** | **Total scores** | **LFT-1** | **LFT-2** | **LFT-3** |
| --- | --- | --- | --- | --- | --- | --- | --- | --- |
| N2 | Lower | M | 28 | 24 | 3 | 1 | 1 | 1 |
| N3 | Lower | F | 24 | 19 | 8 | 3 | 2 | 3 |
| N7 | Lower | M | 22 | 16 | 8 | 2 | 3 | 3 |
| N8 | Lower | F | 21 | 16 | 7 | 2 | 2 | 3 |
| N10 | Lower | M | 23 | 17 | 9 | 2 | 3 | 4 |
| N12 | Lower | M | 29 | 19 | 7 | 1 | 3 | 3 |
| N13 | Lower | F | 26 | 20 | 9 | 5 | 2 | 2 |
| N14 | Lower | F | 42 | 19 | 11 | 3 | 5 | 3 |
| N15 | Lower | M | 25 | 19 | 12 | 6 | 4 | 2 |
| N18 | Lower | F | 28 | 20 | 7 | 4 | 1 | 2 |
| N23 | Lower | F | 17 | 17 | 10 | 3 | 4 | 3 |
| N24 | Lower | M | 53 | 23 | 8 | 4 | 0 | 4 |
| N25 | Lower | F | 52 | 25 | 4 | 3 | 0 | 1 |
| N27 | Lower | F | 21 | 15 | 12 | 3 | 4 | 5 |
| N29 | Lower | M | 24 | 18 | 11 | 5 | 3 | 3 |
| N30 | Lower | M | 22 | 16 | 11 | 6 | 4 | 1 |
| N32 | Lower | F | 25 | 20 | 12 | 6 | 3 | 3 |
| N33 | Lower | F | 24 | 19 | 9 | 4 | 1 | 4 |
| N35 | Lower | F | 36 | 14 | 11 | 3 | 2 | 6 |
| N38 | Lower | M | 25 | 19 | 12 | 4 | 3 | 5 |
| N1 | Higher | M | 25 | 20 | 15 | 6 | 5 | 4 |
| N4 | Higher | M | 25 | 19 | 18 | 8 | 5 | 5 |
| N5 | Higher | M | 26 | 19 | 20 | 7 | 6 | 7 |
| N6 | Higher | F | 32 | 20 | 15 | 5 | 6 | 4 |
| N9 | Higher | M | 22 | 16 | 14 | 4 | 6 | 4 |
| N11 | Higher | M | 28 | 20 | 14 | 5 | 6 | 3 |
| N16 | Higher | F | 24 | 19 | 18 | 9 | 4 | 5 |
| N19 | Higher | M | 45 | 23 | 16 | 7 | 3 | 6 |
| N20 | Higher | F | 42 | 20 | 13 | 6 | 4 | 3 |
| N21 | Higher | F | 35 | 25 | 14 | 6 | 4 | 4 |
| N22 | Higher | F | 25 | 18 | 14 | 5 | 4 | 5 |
| N26 | Higher | M | 37 | 27 | 16 | 6 | 5 | 5 |
| N28 | Higher | F | 21 | 15 | 14 | 5 | 5 | 4 |
| N31 | Higher | M | 27 | 19 | 17 | 6 | 5 | 6 |
| N34 | Higher | F | 25 | 20 | 18 | 6 | 4 | 8 |
| N36 | Higher | F | 25 | 18 | 17 | 7 | 5 | 5 |
| N37 | Higher | F | 28 | 18 | 13 | 6 | 3 | 4 |
