# Supplementary material for: Left Frontotemporal Region Plays a Key Role in Letter Fluency Task-Evoked Activation and Functional Connectivity in Normal Subjects: A Functional Near-Infrared Spectroscopy Study
Source: Front Psychiatry. 2022 May 20;13:810685. doi: 10.3389/fpsyt.2022.810685 (PMC9205401; doi:10.3389/fpsyt.2022.810685)
Supplement: Supplementary file 4 [file Data_Sheet_1.docx]

Supplementary Material

# Supplementary Data

**Instrument**

The hemodynamic changes over the frontotemporal regions were measured using a 52-channel NIRS instrument (ETG-4000; Hitachi Medical Co., Tokyo, Japan). Each paired source-detector was placed at a distance of 3.0 cm. We set the 3 × 11 cell probe with its lowest line at Fp1–Fp2 based upon the EEG international 10–20 system, and extended it laterally to T3 on the left and T4 on the right. Two near-infrared wavelengths (695nm and 830 nm) were used with a 10Hz sampling rate. Most emitted light was reflected back after travelling a short distance in the brain tissue. Brain activity was measured by calculating the light absorbed by the underlying hemoglobin.

**Letter/Phonemic fluency task**

All participants received the Chinese version of the letter fluency task (LFT) with a NIRS probe applied over the anterior head region. Subjects were instructed to gaze at a cross on the screen positioned at a distance of approximately one meter in front of them. They were further asked to stay alert and remain as still as possible for the duration of the task. A cue using a particular Chinese syllable was given with an audible instruction (e.g., "ㄆ", which corresponds to /p/ in the International Phonetic Alphabet), and then the participant was asked to generate as many words beginning with that syllable as possible over a span of 20 seconds (1). A second syllable was then given, and finally a third. Thus, the three sections of the task were completed within one minute. The participant was instructed to repeatedly count from one to five for 30 seconds before the task and for 90 seconds after the task in order to establish baseline readings. Prior to commencing the task and recording the data, the protocol was described in detail to the participants and then practiced once to ensure that it was properly understood. The number of produced words in each of the three sections of the task were recorded and summed, representing the subjects’ LFT scores.

**Data analysis**

We extracted the hemoglobin signal changes during the 60-second task period and analyzed them using MATLAB R2020a (MathWorks, Natick, MA, USA). The NIRS instrument generated two types of values regarding concentration change, oxygenated hemoglobin (HbO) and deoxygenated hemoglobin (HbR). HbO reflects oxygen inflow related to brain activity, while HbR represents oxygen consumption by the tissue (2, 3). HbO has a better BOLD signal correlation, a higher signal-to-noise ratio, and is a better representative of functional connectivity than HbR (2). Therefore, we selected only HbO signals for analysis.

**Proportional network thresholding**

The edges were selected according to the specific percentage of the strongest connections determined by the correlation r values. Because negative correlations were present when the threshold exceeded 0.4, we only tested the proportional threshold from 0.1 to 0.35 at every 0.05 increment. The results of the four graph parameters are depicted in **Figure 3-6**. Proportional thresholding ensures the same network size for comparison, while allowing for a more consistent result when the comparison depends on node degree (4). Each parameter showed the similar trend when using the threshold values from 0.1 to 0.35. Because the proportional threshold of 0.3 had the smallest p value amongst all parameters, we chose 0.3 as the network threshold value for subsequent correlation analysis.

Fifty channels were analyzed after excluding the midline channels. The connections to itself were also excluded. To avoid a double count of the channel pairs, the connections were divided by 2. Then, only the top 30% strongest connections were selected. The total connection number was: (2500-50)/2×0.3= 367.5. In total, there were 367 connections selected in both groups for network construction.

# Supplementary Tables

**Supplementary Table 1.** The relationship between the power of each brain region and LFT performance.

**Supplementary Table 2.** Channel location of our near-infrared spectroscopy.

**Supplementary Table 3.** The detail of characteristics of each subject.

**Reference**

1. Huang C-J, Chou P-H, Wei H-L, Sun C-W. Functional connectivity during phonemic and semantic verbal fluency test: a multichannel near infrared spectroscopy study. *IEEE Journal of Selected Topics in Quantum Electronics*. (2016)22:43-8. doi: [10.1109/JSTQE.2015.2503318](https://doi.org/10.1109/JSTQE.2015.2503318)

2. Nguyen T, Kim M, Gwak J, Lee JJ, Choi KY, Lee KH, et al. Investigation of brain functional connectivity in patients with mild cognitive impairment: A functional near-infrared spectroscopy (fNIRS) study. *J Biophotonics*. (2019) 12:e201800298. doi: [10.1002/jbio.201800298](https://doi.org/10.1002/jbio.201800298)

3. Wolf U, Toronov V, Choi JH, Gupta R, Michalos A, Gratton E, et al. Correlation of functional and resting state connectivity of cerebral oxy-, deoxy-, and total hemoglobin concentration changes measured by near-infrared spectrophotometry. *J Biomed Opt*. (2011) 16:087013. doi: [10.1117/1.3615249](https://doi.org/10.1117/1.3615249)

4. Garrison KA, Scheinost D, Finn ES, Shen X, Constable RT. The (in)stability of functional brain network measures across thresholds. *NeuroImage*. (2015) 118:651-61. doi: [10.1016/j.neuroimage.2015.05.046](https://doi.org/10.1016/j.neuroimage.2015.05.046)
