# Supplementary material for: Left Frontotemporal Region Plays a Key Role in Letter Fluency Task-Evoked Activation and Functional Connectivity in Normal Subjects: A Functional Near-Infrared Spectroscopy Study
Source: Front Psychiatry. 2022 May 20;13:810685. doi: 10.3389/fpsyt.2022.810685 (PMC9205401; doi:10.3389/fpsyt.2022.810685)
Supplement: Supplementary file 5 [file Data_Sheet_2.docx]

**Figure legend**

**Supplementary Figure 1**

The timeline of the task performance.

**Supplementary Figure 2**

**Demonstration of the effect of preprocessing (band-pass filtering, motion correction, detrend) and the bad channels identification**

The signal processing of the 52 channels from one of the subjects was demonstrated as below. Initially, the original HbO concentrations from NIRS instrument were extracted (**left**). The outliers, which were marked by the red lines (channel 2 and channel 4), were identified by the two neurologists. Then, they were excluded for further analysis as the bad channels (**middle**). Later, serials of preprocessing were applied, including band-pass filter, motion correction, and detrend. Finally, the signals were transformed into the smoother configuration (**right**).
