# Supplementary figures and images for: Left Frontotemporal Region Plays a Key Role in Letter Fluency Task-Evoked Activation and Functional Connectivity in Normal Subjects: A Functional Near-Infrared Spectroscopy Study
Source: Front Psychiatry. 2022 May 20;13:810685. doi: 10.3389/fpsyt.2022.810685 (PMC9205401; doi:10.3389/fpsyt.2022.810685)

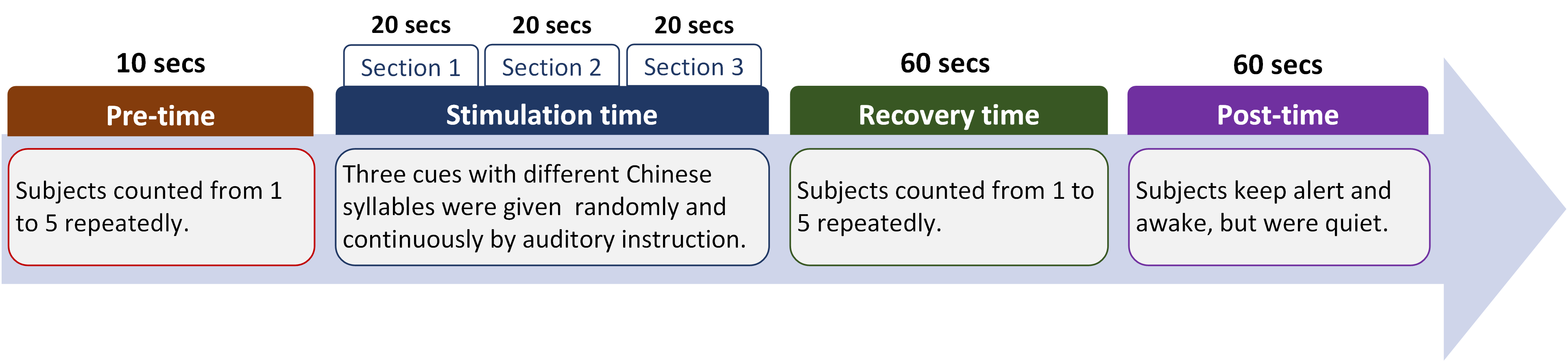

Supplement: Supplementary file 6 [file Image_1.tiff]

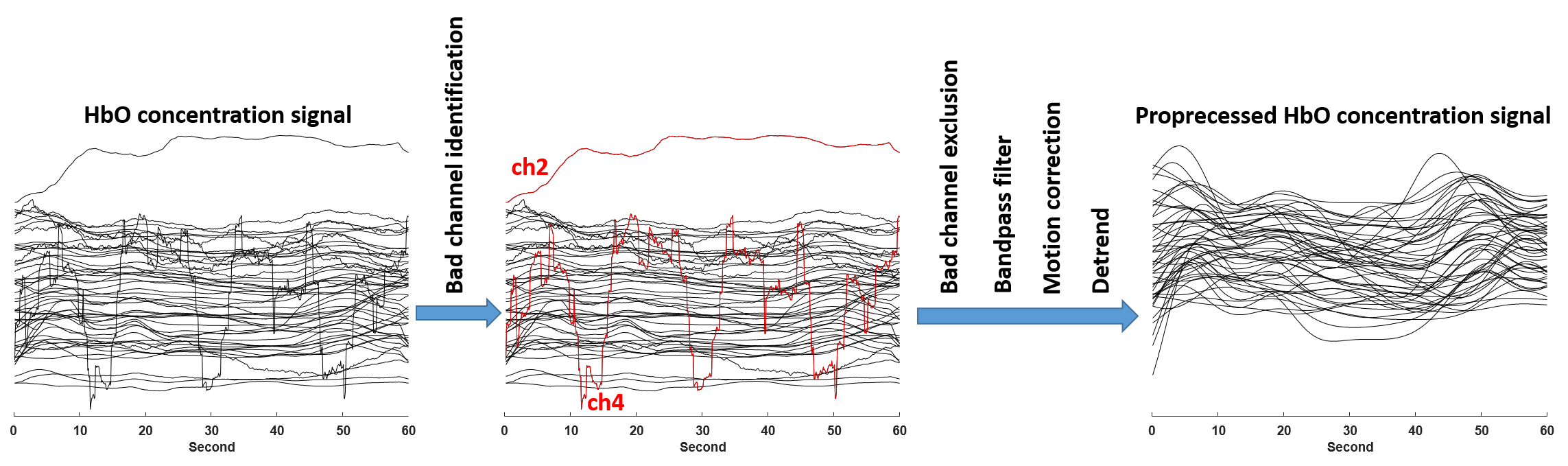

Supplement: Supplementary file 7 [file Image_2.tiff]
